# Correlating transcription and protein expression profiles of immune biomarkers following lipopolysaccharide exposure in lung epithelial cells

**DOI:** 10.1371/journal.pone.0293680

**Published:** 2024-04-23

**Authors:** Daniel E. Jacobsen, Makaela M. Montoya, Trent R. Llewellyn, Kaitlyn Martinez, Kristen M. Wilding, Kiersten D. Lenz, Carrie A. Manore, Jessica Z. Kubicek-Sutherland, Harshini Mukundan

**Affiliations:** 1 Chemistry Division, Los Alamos National Laboratory, Los Alamos, New Mexico, United States of America; 2 Analytics, Intelligence and Technology Division, Los Alamos National Laboratory, Los Alamos, New Mexico, United States of America; 3 Theoretical Division, Los Alamos National Laboratory, Los Alamos, New Mexico, United States of America; University of Illinois, UNITED STATES

## Abstract

Universal and early recognition of pathogens occurs through recognition of evolutionarily conserved pathogen associated molecular patterns (PAMPs) by innate immune receptors and the consequent secretion of cytokines and chemokines. The intrinsic complexity of innate immune signaling and associated signal transduction challenges our ability to obtain physiologically relevant, reproducible and accurate data from experimental systems. One of the reasons for the discrepancy in observed data is the choice of measurement strategy. Immune signaling is regulated by the interplay between pathogen-derived molecules with host cells resulting in cellular expression changes. However, these cellular processes are often studied by the independent assessment of either the transcriptome or the proteome. Correlation between transcription and protein analysis is lacking in a variety of studies. In order to methodically evaluate the correlation between transcription and protein expression profiles associated with innate immune signaling, we measured cytokine and chemokine levels following exposure of human cells to the PAMP lipopolysaccharide (LPS) from the Gram-negative pathogen *Pseudomonas aeruginosa*. Expression of 84 messenger RNA (mRNA) transcripts and 69 proteins, including 35 overlapping targets, were measured in human lung epithelial cells. We evaluated 50 biological replicates to determine reproducibility of outcomes. Following pairwise normalization, 16 mRNA transcripts and 6 proteins were significantly upregulated following LPS exposure, while only five (CCL2, CSF3, CXCL5, CXCL8/IL8, and IL6) were upregulated in both transcriptomic and proteomic analysis. This lack of correlation between transcription and protein expression data may contribute to the discrepancy in the immune profiles reported in various studies. The use of multiomic assessments to achieve a systems-level understanding of immune signaling processes can result in the identification of host biomarker profiles for a variety of infectious diseases and facilitate countermeasure design and development.

## Introduction

The host innate immune response is the body’s first line of defense against pathogens [1, 2]. Human innate immune receptors recognize conserved signatures on pathogens known as pathogen associated molecular patterns (PAMPs), which are important signaling molecules released by pathogens during infection [3, 4]. These molecules bind host cell pattern recognition receptors (PRRs) typically found on endothelial, epithelial, or tissue-resident immune cells such as macrophages and dendritic cells triggering a signal cascade that upregulates expression of cytokines and chemokines. For example, Toll-Like-Receptor 4 (TLR4) recognizes lipopolysaccharide (LPS), a PAMP found in Gram-negative bacteria [5]. The activation of TLR4 triggers the nuclear factor κB (NF-κB) pathway and causes the release of cytokines and chemokines [6]. Cytokines and chemokines signal and recruit specific immune cells to the site of infection [7]. Cytokines include a wide array of molecules including interferons (IFNs), interleukins (ILs), colony-stimulating factors (CSFs), tumor necrosis factors (TNFs), and transforming growth factors (TGFs) [8]. Chemokines are divided into groups by the positioning of their initial cysteine residues: XC, CC, CXC, and CX3C. CC chemokine ligands (CCLs) have two adjoining cysteine residues, CXC ligands (CXCLs) have an amino acid between the cysteine residues [9]. Neutrophils are among the first responders, stimulate leukocyte signaling, phagocytosis, and degranulation [10]. Monocytes differentiate into either macrophages for phagocytosis of extracellular pathogens or dendritic cells (DCs) for antigen presentation [11].

Cultured human cell studies are often used as a model for generating biological insights about specific aspects of a given disease [12]. Cell systems do not entirely replicate the physiological complexity of innate immune recognition, but they provide a reliable and effective model for controlled studies, including the ability to expose cells to individual pathogens or molecules and measure responses over time. However, most cell studies are limited in scope by focusing on a few key cytokine and chemokine targets and avoiding biological variation by using a small number of replicates [13, 14]. Typically, these studies are designed to measure either transcription (mRNA) or protein responses, not both, resulting in limited data sets allowing for a direct comparison of potential mRNA and protein biomarkers of host responses to specific stimuli [15, 16]. Cell regulation is determined by the interplay between mRNA, protein, metabolites and other components of the regulome [17]. Recent studies have highlighted the poor correlation between mRNA and protein profiles in the same experimental system, highlighting the role of other factors such as post-transcriptional machinery in modulating cellular responses [18–21]. In order to begin to explore the correlation between transcriptomic and proteomic profiles in innate immunity, we present a methodical comparison of transcription and protein immune profiles in lung epithelial cells exposed to LPS derived from *Pseudomonas aeruginosa*, a common model for lung response to infection by Gram-negative bacteria [22]. We examined expression of 84 mRNA transcripts and 69 secreted proteins, including 35 overlapping targets, from 5 biological replicates. Supernatants for protein measurements and cell extracts for mRNA measurements were taken at the same time from the same cells, providing a paired examination of mRNA and protein levels. This is a first step towards developing a multiomic approach to understanding innate immune signaling and identifying host biomarker profiles to diagnose and detect infectious diseases.

## Materials and methods

### Cell culture and LPS exposure

Human lung epithelial A549 cells were obtained from American Type Culture Connection (ATCC; CCL-185) and cultured in Kaighn’s Modified Ham’s Formulation F-12 Media (ThermoFisher Scientific; 21127022) with 10% fetal bovine serum (Sigma Aldrich; F2442-500ML) and 1% v/v penicillin/streptomycin (ThermoFisher Scientific; 15140122). Two stocks (frozen at passage #4) were grown separately for a single passage, then split into 6 separate cultures each (total of 12). These 12 flasks, referred to as lineages, were grown for an additional passage to increase biological variation. Over the course of 3 weeks, 5 passages of each cell line were cultured in 24-well plates, with each of the 12 lineages cultured in 2 wells. The night before adding to cells, LPS from *P*. *aeruginosa* suspended in water (Sigma Aldrich; L9143-25MG) was sonicated and diluted to 10 μg/mL in fresh media and stored overnight at 4°C. Once cells reached >80% confluence, the media in one well of each lineage was replaced with media prewarmed to 37°C containing 10 μg/mL LPS (treated), while the other well received fresh, prewarmed media without LPS (untreated). After 24 hours, the supernatant was removed and stored at –80°C until protein analysis and RNA was extracted from the cells using the Qiagen RNeasy Mini Kit (Qiagen; 74106). Post-extraction, RNA concentration was measured using the Qubit RNA Broad Range Assay Kit (ThermoFisher Scientific; Q10211) and stored at –80°C until further use.

### Transcript expression analysis

500 ng RNA from each sample was used as input for first strand synthesis (Qiagen; 330404). After first strand synthesis, samples were run on 96-well Human Cytokines and Chemokines RT2 Profiler PCR Arrays (Qiagen; PAHS-150ZC-24) using the RT2 qPCR Master Mix (Qiagen; 330529). The Cytokine and Chemokine RT2 array measured RNA levels of 84 cytokines and chemokines, 5 housekeeping genes, and included 7 control wells. mRNA expression was measured for 100 replicates, 50 LPS-treated and 50 matching untreated controls from the same lineage and plate. The plates were run on an Applied Biosystems StepOnePlus Real-Time PCR Thermocycler (ThermoFisher Scientific; 4376600) using the following protocol: 95°C for 10 min; 40 cycles: 95°C for 15 sec; 60°C for 1 min. Cycle threshold (C_T_) values were used as input for data analysis and normalized to the housekeeping gene beta-2 microglobulin (B2M). B2M was chosen as the housekeeping gene due to it having the least-significant difference between treated and untreated samples (see S1 Fig). Fold change, also commonly termed relative quantification or RQ, was calculated using pairwise 2^-ΔΔCT^ for each transcript target *T*, which is the difference in normalized C_T_ between the LPS-treated sample (+) and its untreated control (-) from the same lineage *l* and plate *p*, then averaged across all *N* samples,

$$Fold\;Change_{T}=\;\frac{\sum_{p,l}2^{-\Delta \Delta CT_{T,\;p,\;l}}}{N}$$ (1)

$$2^{-\Delta \Delta CT_{T,\;p,\;l}}=2^{-\left(\Delta CT_{T,p,l,+}-\Delta CT_{T,p,l,-}\right)}$$ (2)

$$\Delta CT_{T,p,l,t}=CT_{T,p,l,t}-CT_{B2M,p,l,t}\;\text{for}\;t=+,-$$ (3)

### Protein analysis

Supernatants were analyzed for secreted proteins using the abcam FirePlex Discovery Human Cytokine and Chemokine panel (abcam; ab243551), which measures protein levels of 69 cytokines and chemokines and a negative control used for normalization. 35 of the proteins measured had their corresponding mRNA measured in transcript analysis above, providing direct comparison between mRNA and protein levels. Prior to starting the FirePlex protocol, supernatants were centrifuged at 2000 x g for 15 minutes to remove debris. Samples were processed using the vacuum filtration plate option of the FirePlex protocol, vacuum filtration manifold (abcam; ab204067), and analyzed on a Beckman Coulter CytoFLEX S Flow Cytometer (Beckman Coulter; C09766). The cytometer measured FITC (gain 70), PE (gain 50), and PC5.5 (gain 15), using a flow rate of 60 μL/min with samples run for 3 minutes or 5,000 events of FITC signal ≥10,000. Raw output from the flow cytometer was analyzed using the Firefly Analysis Workbench software from abcam (https://www.fireflybio.com), which generated measurements of signal for each sample and protein. Known standards for each protein were also run in the FirePlex assay and used to convert signal measurements to concentrations in pg/mL. For comparative analysis, values above the maximum signal of the standard curve were capped at the maximum value and values below the minimum signal of the standard curve were capped at the minimum. Normalized protein concentrations $\left[\bar{P}\right]$ were obtained by dividing each pg/mL value [*P*] by the control ratio value *CR*, which was the value of the negative control [*NC*] for that sample divided by the average negative control value across all samples. Fold-change for each protein target *T* was calculated by pairwise division of normalized pg/mL treated values by untreated values, then pairwise-calculated fold-change was averaged across plates *p* and lineages *l*.


$$Fold\;Change_{\;T}=\frac{\sum_{p,l}\frac{{\left[\bar{P}\right]}_{T,p,\;l,+}}{{\left[\bar{P}\right]}_{T,p,\;l,-}}}{N}$$ (4)

$${\left[\bar{P}\right]}_{T,p,l,t}=\frac{{\left[P\right]}_{T,p,l,t}}{CR_{p,l,t}}\;\text{for}\;t=+,-$$ (5)

$$CR_{p,\;l,t}=\frac{{\left[NC\right]}_{p,\;l,t}}{\frac{\sum_{p,\;l,t}{\left[NC\right]}_{p,\;l,t}}{N}}\;\text{for}\;t=+,-$$ (6)

Some protein samples detected no particles, meaning no FirePlex particles for a protein were detected by the flow cytometer for that individual sample. As this non-detection does not indicate absence of the protein in the sample but is the result of dropouts from random sampling, these data cannot be considered true zeros. As a result, these samples were left out of the analysis, as well as the analogous treated/untreated sample from the same lineage and plate for pairwise comparisons.

### Statistical analysis

Statistical analyses were performed using python 3.8.8 [23]. Significance of mRNA expression was determined by pairwise Student’s t-test between B2M-normalized C_T_ values (Δ*CT*_*T*,*p*,*l*,*t*_ values in Eq 3 above) of LPS-treated samples and their corresponding untreated control. Significance in protein expression was calculated by pairwise Student’s t-test between LPS-treated samples and their corresponding untreated control using data normalized with negative control of each sample and converted to pg/mL. These values were chosen so that a pairwise Student’s t-test could be used, reflecting the paired treated/untreated sample of each plate and lineage.

## Results and discussion

### Generation of reproducible transcription and protein expression profiles following LPS exposure in lung epithelial cells

**Fig 1:**
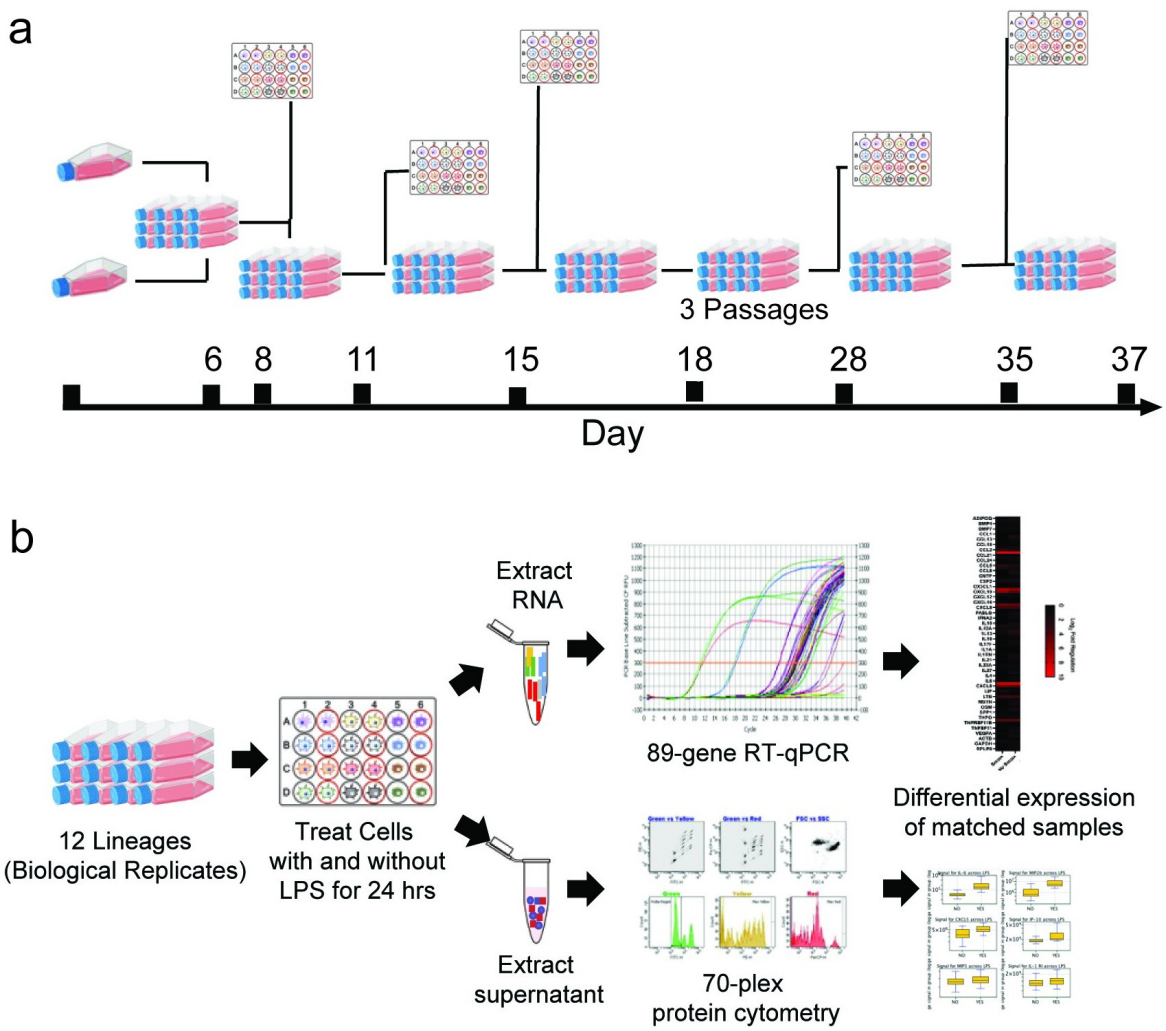
Schematic of experimental workflow evaluating mRNA and protein expression of lung epithelial cells following LPS exposure. a) Timeline of experiments. Two cell stocks at the same passage number were thawed and cultured. After one passage, stocks were split into six flasks each (12 total lineages). At five timepoints over 11 passages, samples were collected from each lineage. b) At each of the five time points, each lineage was seeded in two wells of a 24-well plate. One well was treated with 10 μg/mL LPS diluted in media, the other received fresh media without LPS (untreated), and all were incubated for 24 hours at 37°C with 5% CO_2_ prior to harvesting of mRNA and supernatant. mRNA levels were evaluated using reverse-transcription quantitative polymerase chain reaction (RT-qPCR) and protein levels were measured in supernatants using immunoassays. Figure created with BioRender.com.

Human lung epithelial A549 cells were selected for this study. A549 cells are not a comprehensive model for lung infection but have been used often for simple controlled studies of LPS stress on cells [14, 24, 25]. Biological replicates of A549 cells in this study were derived from twelve lineages, produced from two frozen stocks at the same passage number, then split into six new flasks each and harvested at five time points (Fig 1a). At the five time points, cells were seeded in 24-well plates, with each of the twelve lineages receiving two wells (Fig 1b). Near confluency, one of the wells of each lineage received media containing 10 μg/mL LPS (treated), and the other well received fresh media (untreated), and then both were incubated for 24 hours prior to harvesting. 10 μg/mL LPS was found to be enough to stimulate an immune response without reducing cell viability (S2 Fig and [24]). Incubation of 24 hours was chosen to provide more consistent results, as dynamic changes in protein levels make the early timepoints more variable [26]. For harvesting, supernatant of each sample was used for protein analysis and the cells were harvested for mRNA analysis (see S1 Methods in S1 File). Each biological replicate had two wells used for treated and untreated controls, so pairwise analysis was used to compare LPS-treated expression profiles relative to the matched untreated control prior to averaging across all samples. This was necessary to reduce bias caused by lineage and plate (S3 Fig). In this study, 50 biological replicates with matched treated and untreated controls were analyzed for mRNA and protein expression to identify biomarkers of LPS exposure.

### Biomarkers of LPS exposure in lung epithelial cells include cytokines and chemokines involved in cellular recruitment and pro-inflammatory responses

**Fig 2:**
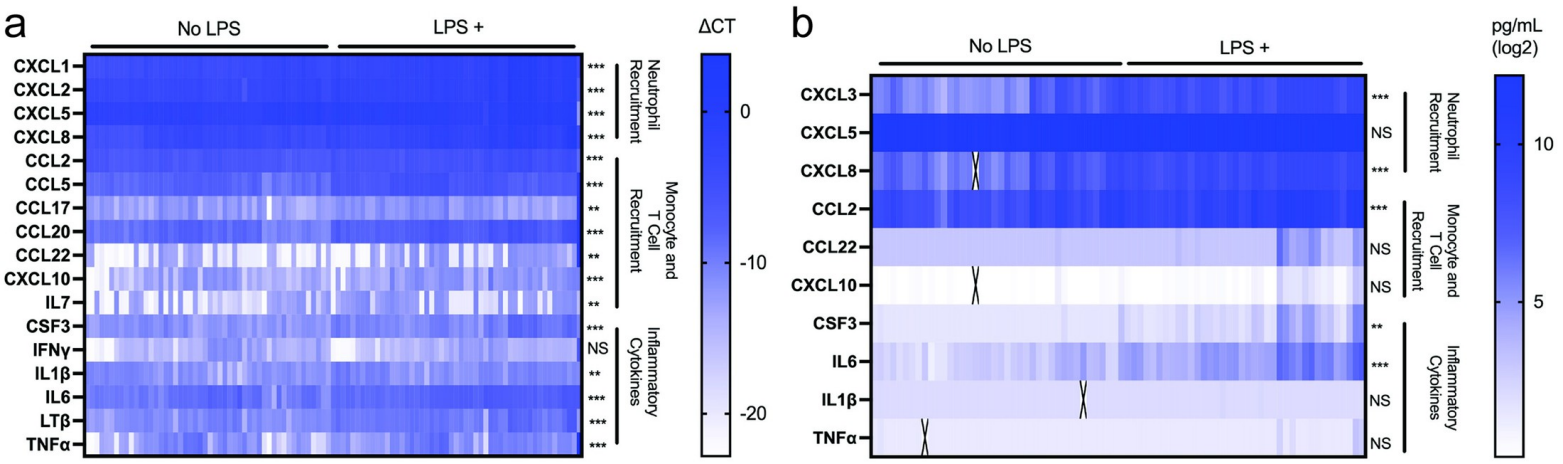
mRNA and protein expression profiles in response to LPS exposure. Each column represents an individual biological replicate for (a) mRNA and (b) protein analysis. mRNA values are displayed as ΔC_T_ values following normalization to the housekeeping gene B2M C_T_ value (see S1 Methods in S1 File). Protein values are displayed as log2 pg/mL following normalization to negative control (see S1 Methods in S1 File). Student’s paired t-tests were performed comparing LPS-treated to untreated samples of the same plate and lineage (***p<0.001; **p<0.01; NS, not significant). IL, interleukin; CCL, chemokine ligand; CXCL, chemokine (C-X-C motif) ligand; TNFα, tumor necrosis factor alpha; IFNγ, interferon gamma; LTβ, lymphotoxin beta; CSF, colony stimulating factor.

Expression levels of human cytokines and chemokines were evaluated using commercial kits including 84 mRNA and 69 protein targets. In response to LPS treatment, 16 cytokines and chemokines showed significant upregulation in mRNA expression (Fig 2a). Four of them (CXCL1, CXCL2, CXCL5, and CXCL8) are chemokines involved in neutrophil recruitment [27]. Seven of them (CCL2, CCL5, CCL17, CCL20, CCL22, CXCL10, and IL7) are involved in monocyte and T cell recruitment [28–34]. T cells are recruited to early sites of inflammation including CD4^+^ T helper (T_H_) cells that produce cytokines to orchestrate coordinated immune responses [35]. Different cell subsets target different types of pathogens, with T_H_1 cells targeting intracellular viruses and bacteria, T_H_2 cells targeting extracellular parasites, and T_H_17 cells targeting extracellular bacteria. Naïve CD4^+^ T cells may also divide into regulatory T cells (T_reg_) that suppress immune responses. CCL17 and CCL22 have high selectivity towards T_H_2 cells [36], which are known to be promoted in response to LPS [37]. CCL20 is primarily involved in recruitment of T_H_17 effector T cells. CXCL10 primarily affects T_H_1 effector T cells and has been more closely associated with viral infections [38]. CCL2 has been shown to have a significant effect on monocytes, inducing chemotaxis, differentiation into macrophages, and adhesion to endothelium.

Five pro-inflammatory cytokines (CSF3, IL1β, IL6, lymphotoxin β (LTβ), and TNFα) were also significantly upregulated. CSF3 is a potent stimulator of myeloid cell proliferation and preferentially directs myeloid progenitors into neutrophil differentiation [39]. LTβ signaling is associated with tertiary development of lymphoid tissue [40] and has been linked with neutrophil inflammation [41]. IL6, TNFα, and IL1β are cytokines with systemic, wide-ranging effects, and increased expression following LPS exposure has been shown previously [14]. Additionally, increased levels of these cytokines have been associated with increased severity of respiratory distress [42].

Protein expression showed only six significantly upregulated proteins (Fig 2b). CXCL5 signal was saturated in the FirePlex assay, but enzyme-linked immunosorbent assay (ELISA) results showed a significant increase from LPS treatment (See S1 Methods and S4 Fig). Five of the six significantly upregulated proteins (CCL2, CSF3, CXCL5, CXCL8, and IL6) were upregulated in both mRNA and protein levels. The other protein, CXCL3 [43], is involved in neutrophil recruitment. CXCL3 is part of a subset of chemokines known as Growth Related Oncogene (GRO) chemokines, along with CXCL1 and CXCL2. In this study, CXCL1 and CXCL2 were only investigated in mRNA while CXCL3 was only investigated in protein due to the composition of the commercial panels used. All three were significantly upregulated indicating the neutrophil recruitment pathway as a potential biomarker for LPS exposure at both the transcript and protein expression levels.

### Differences in cytokine and chemokine mRNA and protein expression profiles can be used as biomarkers for LPS exposure

**Fig 3:**
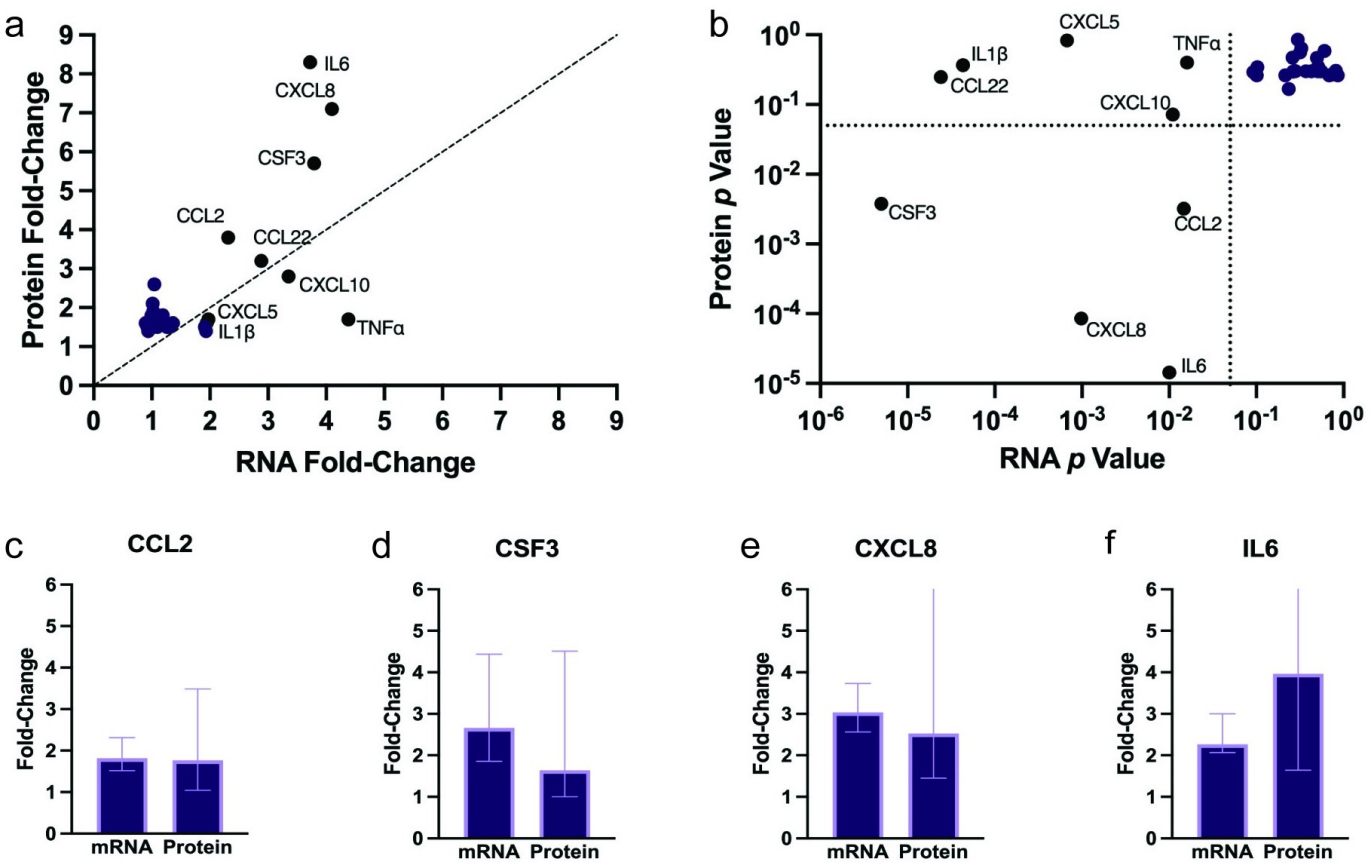
LPS exposure results in discrepancies in mRNA and protein expression profiles of key cytokines and chemokines. (a) Fold-changes in mRNA (x-axis) versus protein (y-axis) expression comparing LPS-treated to untreated controls. Dashed line indicates equivalent RNA and protein fold-changes. (b) *p*-values from pairwise Student’s t-test for RNA (x-axis) versus protein (y-axis) fold-changes. Dashed lines indicate *p* = 0.05. Cytokines and chemokines without significant changes in expression of mRNA and/or protein were not displayed. Significance was calculated using paired Student’s t-test comparing LPS-treated cells to untreated controls from the same lineage and plate. Individual plots comparing fold-changes in cytokines and chemokines significantly upregulated in both mRNA and protein expression for (c) CCL2, (d) CSF3, (e) CXCL5, (f) CXCL8, and (g) IL6. Values represent median signal and bars represent 95% confidence interval. Significance was calculated using pairwise-Student’s t-test matching mRNA and protein fold changes from the same lineage and plate. CXCL5 values represent values obtained via ELISA data (see S1 Methods and S4 Fig). No significant difference was found between mRNA and protein levels. IL, interleukin; CCL, chemokine ligand; CXCL, chemokine (C-X-C motif) ligand; TNF, tumor necrosis factor alpha; CX3CL, (C-X3-C) chemokine ligand; CSF, colony stimulating factor.

Of the 84 mRNA and 69 protein targets, 35 overlapped and were evaluated in both data sets for differences in fold-change (Fig 3a) and significance (Fig 3b). Five cytokines and chemokines were significantly upregulated in both mRNA and protein: CCL2 (Fig 3c), CSF3 (Fig 3d), CXCL5 (Fig 3e), CXCL8/IL8 (Fig 3f), and IL6 (Fig 3g). For all five cytokines and chemokines, the average fold-change of mRNA and protein was identical. These cytokines and chemokines promote neutrophil differentiation (CSF3) and recruitment (CXCL5 and CXCL8), monocyte recruitment (CCL2), and general inflammation (IL6). All five can be used as biomarkers for LPS exposure at both the transcript and protein level.

Four cytokines and chemokines were significantly upregulated in mRNA measurements, but not in protein (IL1β, TNFα, CXCL10, and CCL22). All four have regulatory mechanisms with additional checkpoints between mRNA expression and mature protein secretion. Two cytokines with mRNA and protein discrepancies, IL1β and TNFα, are two of the most potent immune stimulators with systemic effects [44, 45]. As a result, both proteins have several checkpoints before secretion. LPS-binding to TLR4 can initiate IL1β transcription and translation into a pro-IL1β form that remains in the cytosol [46]. The cleavage of pro-IL1β into its secreted form is dependent on the inflammasome signaling pathway [47]. Previous studies indicate that LPS at 10 μg/mL, the concentration used in this study, does not induce inflammasome signaling [24], supporting the absence of IL1β protein upregulation in our experiments. TNFα is produced in an inert, trimeric form that resides on the cellular membrane. This form is cleaved by a disintegrin and metalloprotease 17 (ADAM17; also known as TNFα converting enzyme, TACE) [48]. Previous work in gastric epithelial cells showed that ADAM17 cleavage of membrane-bound TNFα can be induced by Epidermal Growth Factor Receptor (EGFR) activation by *Helicobacter pylori* or by exogenous TNFα [49]. Another study found that intestinal epithelial cells do not produce TNFα following LPS exposure, however gut macrophages release TNFα and stimulate secretion [50].

The chemokine CXCL10 has been shown to require signals from TH_1_ effector T cells for secretion by epithelial cells [51]. CCL22 secretion by intestinal epithelial cells has been shown to be stimulated by TNFα [52] and has been shown to promote recruitment of TH_2_ cells [53] and T_reg_ cells [54] when secreted by immune cells.

To confirm that the four proteins were not upregulated from LPS treatment, ELISA was performed for these genes and CXCL8 as a positive control in both cell supernatant and cell extract (S4 Fig). ELISA was also performed on CXCL5 using diluted samples, since CXCL5 had saturated signal in the FirePlex assay. Supernatants were used for proteomic analysis because cytokines and chemokines are signaling molecules that must typically be secreted to have an effect. However, intracellular protein levels may provide additional information of cytokines and chemokines not yet secreted or that have been taken up by cells, and so they were examined by ELISA as well. The ELISA results confirmed four proteins were not upregulated in supernatant or cell extract. ELISA analysis of intracellular and extracellular protein levels on six selected genes also suggested that intracellular protein behavior in response to LPS mirrored that of secreted protein behavior.

There is a significant discrepancy between mRNA and protein profiles of cytokines and chemokines at a single time point, as evidenced by our findings. As described above, additional regulation mechanisms may prevent increased mRNA levels from translating to increased secreted protein levels for the five genes with discrepancies in this study. These discrepancies can shed light into mechanisms of LPS action in different cell systems. In particular, the four genes noted above have been shown to be secreted following signaling by immune cells. For biomarker discovery, this indicates that mRNA expression may be more sensitive to pathogens, as protein regulation has additional checkpoints before secretion. This indicates mRNA analysis in early infection can yield a more comprehensive expression profile supporting disease-specific diagnostics.

## Conclusion

Immune responses are a complex interaction of many cell types. Identifying host biomarkers of PAMP or pathogen exposure can be difficult due to this complexity. In this work, we developed a large-scale experimental protocol with 50 biological replicates to investigate the reproducibility of resolution phase immune responses from human lung epithelial cells in response to LPS exposure. We compared expression profiles of cytokines and chemokines for 84 mRNA transcripts and 69 secreted proteins, with 35 overlapping. We found that LPS exposure results in the statistically significant upregulation of 16 mRNA transcripts and only 6 proteins. Furthermore, our work demonstrated four examples of genes whose mRNA expression was significantly upregulated without a corresponding increase in protein. This finding highlights the caution that works should use in assuming that protein upregulation matches mRNA expression upregulation and vice versa. Multiomics approaches can address this problem, as can single omics experiments that focus on biomarkers specific to that omics. In this experiment, we found that transcription analysis provided the more promising means for identifying a unique expression profile for *P*. *aeruginosa* LPS exposure in lung epithelial cells. Future work will determine the feasibility of using human cytokine and chemokine expression levels to distinguish exposure to different PAMPs or pathogens as a means for diagnosing any infection. The complexity of these responses is not readily amenable to manual decoding. Machine learning algorithms can be a powerful tool to analyze these types of datasets and identify immune response expression profiles associated with specific PAMPs or pathogen infections [2, 55, 56].

## Supporting information

S1 Fig Evaluation of mRNA expression of housekeeping genes. Housekeeping genes C_T_ values across all samples for LPS+ and LPS- samples. Effect of LPS treatment (mean difference) and its corresponding p-value were determined for each housekeeping gene. B2M showed the smallest effect size from LPS (mean difference = -0.0897) and largest p-value (p-value = 0.552), indicating it as the best gene to use for normalization.
(TIF)

S2 Fig Cell viability across LPS concentrations. Cell viability, measured as the increase in NucBlue ReadyProbe stain (Hoechst 33342) over a 24-hour period for cells incubated with varying concentrations of LPS. All samples are normalized to the increase seen in cells incubated with no LPS. Error bars represent standard deviation of 3 bioreplicates, which were each determined as the average of 4 technical replicates (4 different wells on a 96 well plate). Line represents best fit using log-scale concentration.
(TIF)

S3 Fig Paired normalization of samples reduces bias caused by date of experiment. Principal component analysis (PCA) of mRNA expression data. (a) Percentage of variance explained by each principal component in mRNA expression data normalized by that sample’s B2M housekeeping gene expression. (b) Pearson r correlation of each principal component of normalized mRNA data with three main variables of experiment, LPS treatment, date of experiment (Plate), and lineage. Principal component (PC) 1 correlates most closely with plate (Pearson r = 0.54) while PC 2 correlates most closely with LPS treatment (Pearson r = 0.61). (c-f) Each dot represents principal component values of (c, d) expression data normalized to that sample’s B2M housekeeping gene expression, (e, f) or pairwise-normalized data of one treated/untreated pair for a given lineage and date. (c) PC 1 and PC 2 of normalized expression data, with colors/numbers indicating date of experiment, with “1” being the first date and “5” being the last date. (d) PC 1 and PC 2 of normalized expression data, with colors indicating LPS treatment of sample. Pairwise-normalized data does not correlate well in PC 1 (13.6% of variance) or PC 2 (11.2% of variance) with either (e) experiment date or (f) lineage. (g) Percentage of variance explained by each principal component in protein data normalized by that sample’s negative control (see S1 Methods in S1 File). (h) Pearson r correlation of each principal component of normalized protein expression data with three main variables of experiment, LPS treatment, date of experiment (Plate), and lineage. PC 5 is the first principal component for which LPS treatment is the strongest correlated category.
(TIF)

S4 Fig ELISA measurements of supernatant and cell extracts. Measured concentrations in pg/mL from matching cell supernatant and cell extract for LPS-treated and untreated A549 cells. Error bars represent standard deviations of 4 biological replicates for supernatants (circles) or cell extracts (triangles). Biological replicate values are the average of three technical replicates, separate wells of a 24-well plate cultured with cells from the same source flask. Student’s paired t-test was performed comparing biological replicates of LPS-treated and untreated samples (*p<0.05; ***p<0.001). IL, interleukin; CCL, chemokine ligand; CXCL, chemokine (C-X-C motif) ligand; TNF, tumor necrosis factor alpha.
(TIF)

S1 Methods Supplemental methods. Explanation of methods used in generating supplemental figures.
(DOCX)
