# Supplementary figures and images for: Correlating transcription and protein expression profiles of immune biomarkers following lipopolysaccharide exposure in lung epithelial cells
Source: PLoS One. 2024 Apr 23;19(4):e0293680. doi: 10.1371/journal.pone.0293680 (PMC11037529; doi:10.1371/journal.pone.0293680)

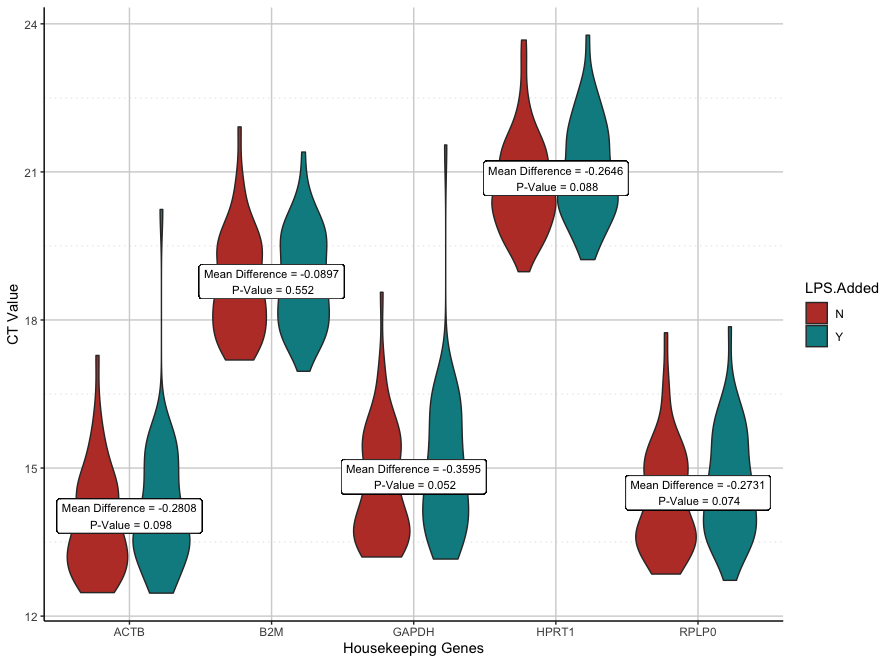

Supplement: S1 Fig — Housekeeping genes CT values across all samples for LPS+ and LPS- samples. Effect of LPS treatment (mean difference) and its corresponding p-value were determined for each housekeeping gene. B2M showed the smallest effect size from LPS (mean difference = -0.0897) and largest p-value (p-value = 0.552), indicating it as the best gene to use for normalization. (TIF) [file pone.0293680.s001.tif]

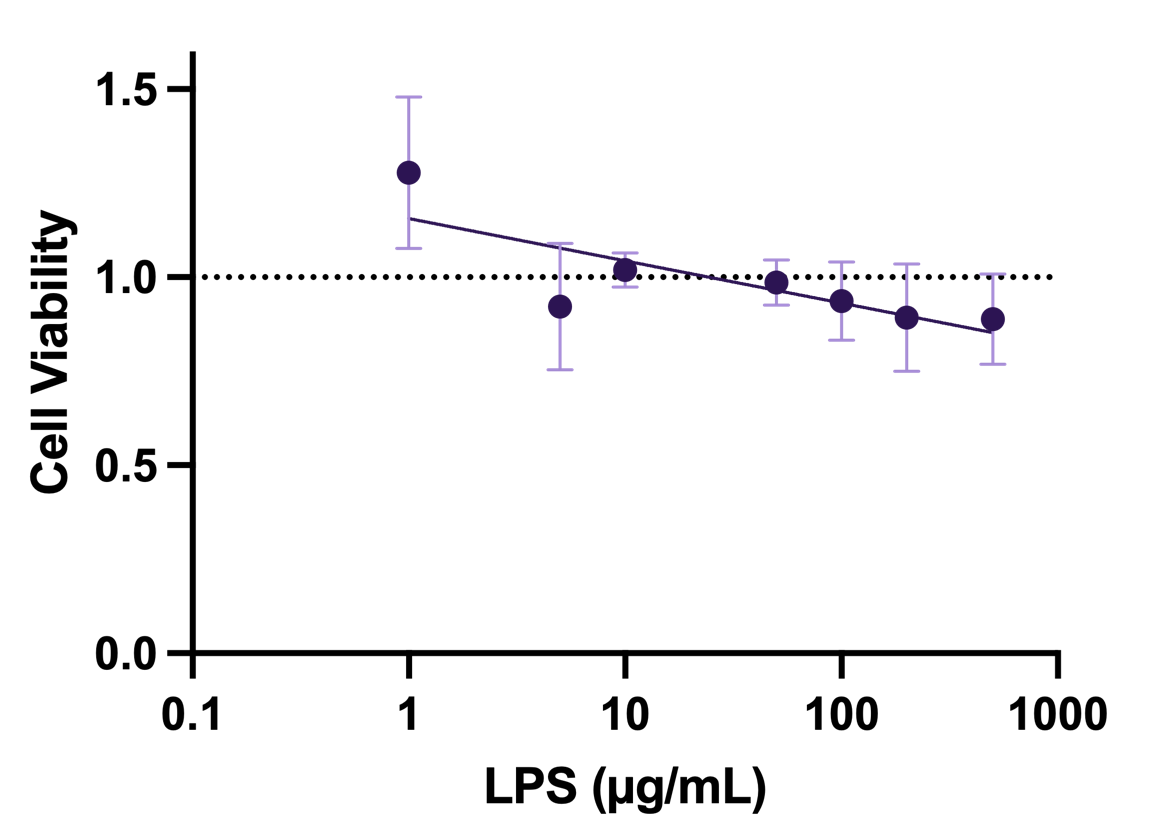

Supplement: S2 Fig — Cell viability, measured as the increase in NucBlue ReadyProbe stain (Hoechst 33342) over a 24-hour period for cells incubated with varying concentrations of LPS. All samples are normalized to the increase seen in cells incubated with no LPS. Error bars represent standard deviation of 3 bioreplicates, which were each determined as the average of 4 technical replicates (4 different wells on a 96 well plate). Line represents best fit using log-scale concentration. (TIF) [file pone.0293680.s002.tif]

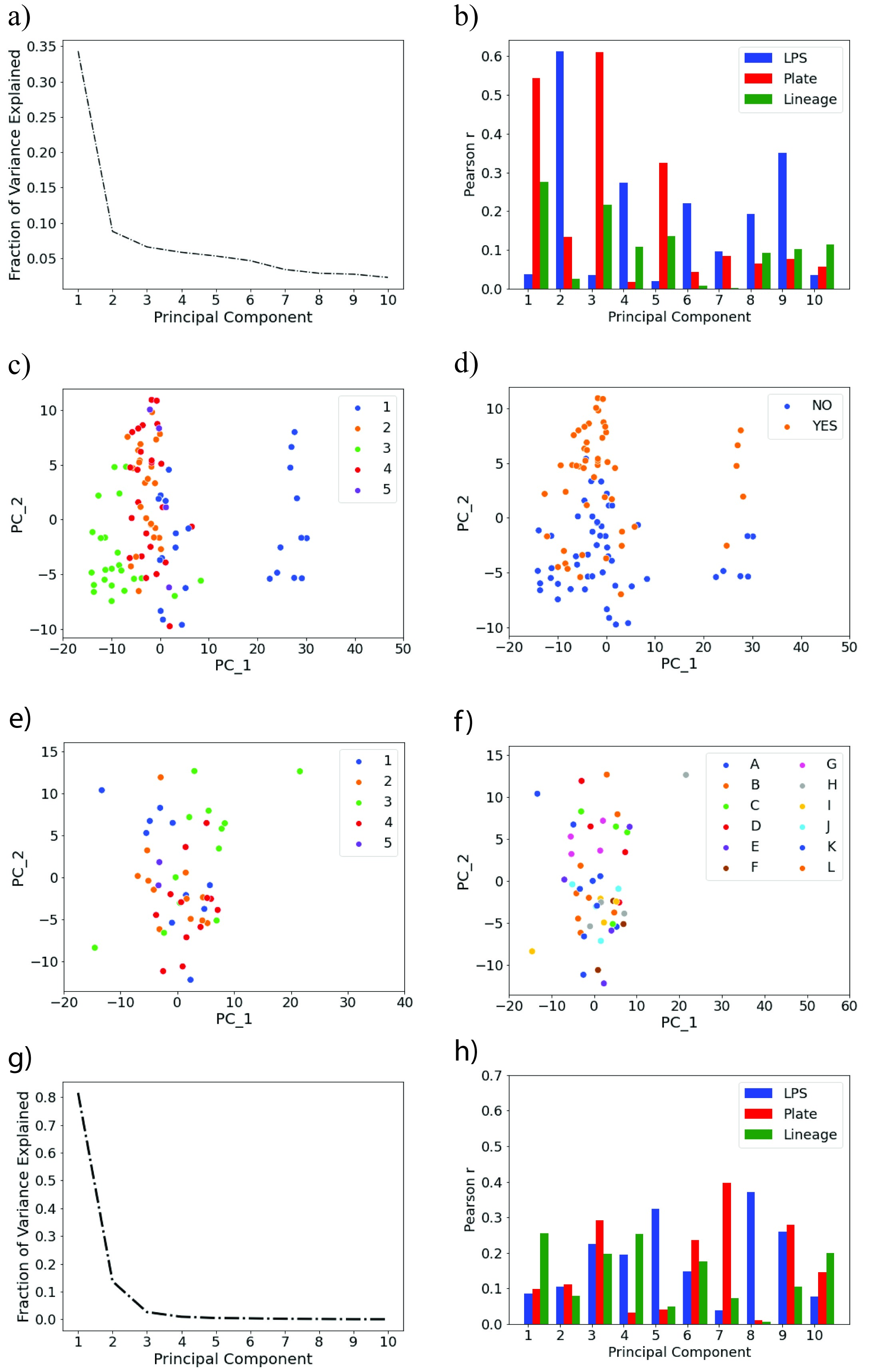

Supplement: S3 Fig — Principal component analysis (PCA) of mRNA expression data. (a) Percentage of variance explained by each principal component in mRNA expression data normalized by that sample’s B2M housekeeping gene expression. (b) Pearson r correlation of each principal component of normalized mRNA data with three main variables of experiment, LPS treatment, date of experiment (Plate), and lineage. Principal component (PC) 1 correlates most closely with plate (Pearson r = 0.54) while PC 2 correlates most closely with LPS treatment (Pearson r = 0.61). (c-f) Each dot represents principal component values of (c, d) expression data normalized to that sample’s B2M housekeeping gene expression, (e, f) or pairwise-normalized data of one treated/untreated pair for a given lineage and date. (c) PC 1 and PC 2 of normalized expression data, with colors/numbers indicating date of experiment, with “1” being the first date and “5” being the last date. (d) PC 1 and PC 2 of normalized expression data, with colors indicating LPS treatment of sample. Pairwise-normalized data does not correlate well in PC 1 (13.6% of variance) or PC 2 (11.2% of variance) with either (e) experiment date or (f) lineage. (g) Percentage of variance explained by each principal component in protein data normalized by that sample’s negative control (see S1 Methods in S1 File). (h) Pearson r correlation of each principal component of normalized protein expression data with three main variables of experiment, LPS treatment, date of experiment (Plate), and lineage. PC 5 is the first principal component for which LPS treatment is the strongest correlated category. (TIF) [file pone.0293680.s003.tif]

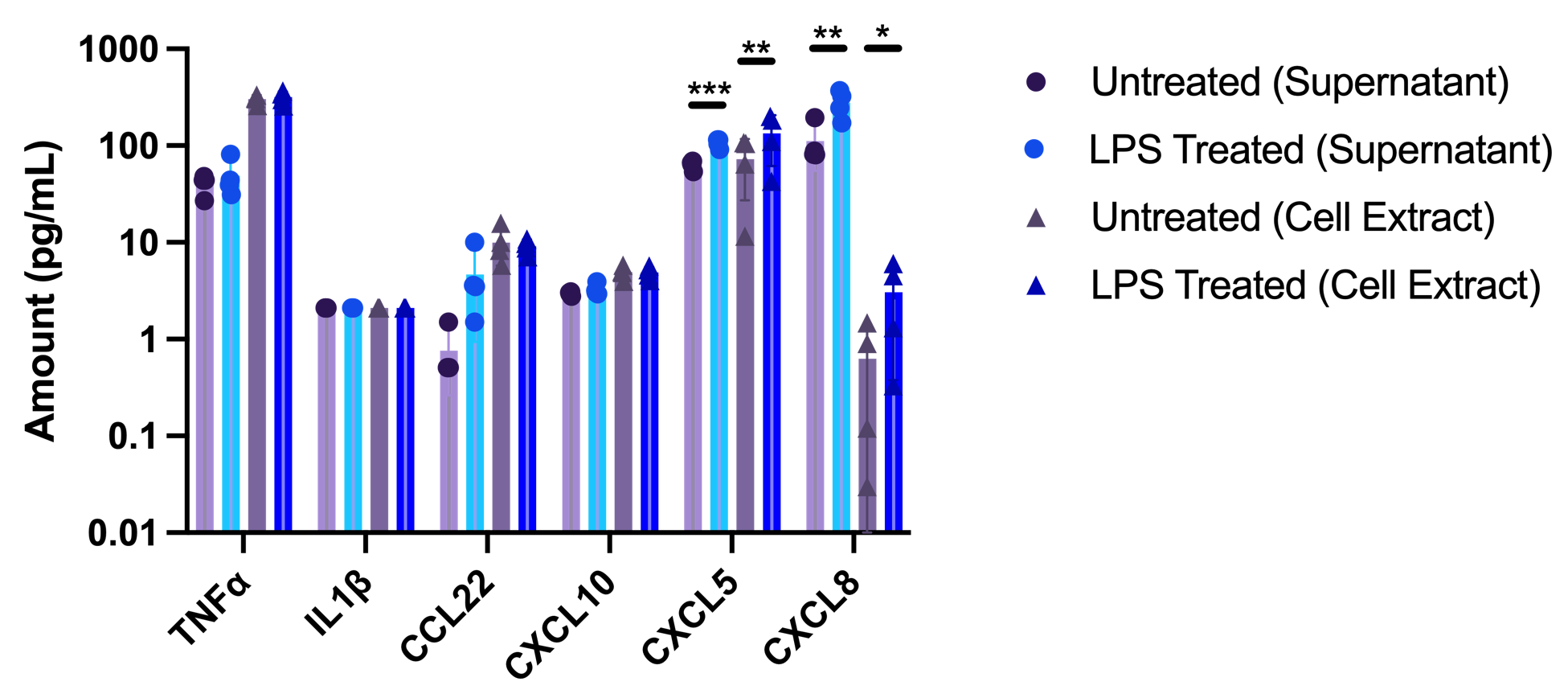

Supplement: S4 Fig — Measured concentrations in pg/mL from matching cell supernatant and cell extract for LPS-treated and untreated A549 cells. Error bars represent standard deviations of 4 biological replicates for supernatants (circles) or cell extracts (triangles). Biological replicate values are the average of three technical replicates, separate wells of a 24-well plate cultured with cells from the same source flask. Student’s paired t-test was performed comparing biological replicates of LPS-treated and untreated samples (*p<0.05; ***p<0.001). IL, interleukin; CCL, chemokine ligand; CXCL, chemokine (C-X-C motif) ligand; TNF, tumor necrosis factor alpha. (TIF) [file pone.0293680.s004.tif]
