# Supplementary material for: Correlating transcription and protein expression profiles of immune biomarkers following lipopolysaccharide exposure in lung epithelial cells
Source: PLoS One. 2024 Apr 23;19(4):e0293680. doi: 10.1371/journal.pone.0293680 (PMC11037529; doi:10.1371/journal.pone.0293680)
Supplement: S1 Methods — Explanation of methods used in generating supplemental figures. (DOCX) [file pone.0293680.s005.docx]

**Supporting Information-Methods**

**Correlating transcription and protein expression profiles of immune biomarkers following lipopolysaccharide exposure in lung epithelial cells**

Daniel E. Jacobsen^1^, Makaela M. Jones^1^, Trent R. Llewellyn^1^, Kaitlin M.L. Martinez^2^, Kristen M. Wilding^3^, Kiersten D. Lenz^1^, Carrie A. Manore^3^, Jessica Z. Kubicek-Sutherland^1,*^, and Harshini Mukundan^1,#a^*

^1^ Chemistry Division, Los Alamos National Laboratory, Los Alamos, New Mexico, USA

^2^ Analytics, Intelligence and Technology Division, Los Alamos National Laboratory, Los Alamos, New Mexico, USA

^3^ Theoretical Division, Los Alamos National Laboratory, Los Alamos, New Mexico, USA

^#a^ Current address: Bioscience Area, Lawrence Berkeley National Laboratory, Berkeley, California, USA

* Corresponding authors:

[jzk@lanl.gov](mailto:jzk@lanl.gov) (J.K.S.) and [harshini@lanl.gov](mailto:harshini@lanl.gov) (H.M.)

**Cell viability studies**

To test the effect of lipopolysaccharide (LPS) on cell viability, cells were seeded onto a 96 well plate and grown to approximately 50% confluency. Cells were then stained using NucBlue Live ReadyProbes (ThermoFisher Scientific; R37605) following manufacturer protocol, and fluorescence measured in the DAPI channel on a BioTek Cytation 7 Cell Imaging Multimode Reader (Agilent). Cell media was then removed and replaced with LPS, prepared as previously noted, of varying concentrations. Cells were incubated in LPS-containing media overnight, then stained with NucBlue Live ReadyProbes and measured in the Cytation 7. Viability was measured as the ratio increase in fluorescence (24 hours/0 hours) for that well, normalized by the increase in fluorescence for wells containing media with no LPS. 3 bioreplicates were performed at each concentration, and each bioreplicate is the average of the increase between 4 technical replicates. 4 technical replicates were performed for each bioreplicate at each LPS concentration, by seeding 4 wells from the same flask onto the plate. Bioreplicates represent different source flasks.

**Analysis of bias and pairwise normalization**

Principal Component Analysis (PCA) was used to evaluate the effects of lineage and date of harvest on the data. PCA identifies the sources of variation in large matrices and ranks them, with principal component (PC) 1 corresponding to the largest source of variation, and PC 2 being less (S3a Fig) [1]. In normalized mRNA expression data, PC 1, corresponding to 34.3 % of the variation, correlated best with the date of experiment (S3b, S3c Figs; Pearson r correlation coefficient=0.54). In contrast, PC 2 corresponding to 8.8 % of the variation correlated best with LPS treatment (S1b, S3d Figs; Pearson r correlation coefficient=0.61). This indicates a large bias in the mRNA data corresponding to the experiment date. This bias was removed by evaluating the differential expression between LPS-treated samples and the untreated controls of the same lineage and date of experiment (S3e and S3f Fig), therefore pairwise-normalized data was used in this study. A similar trend occurred in the protein data. Although most of the variance was explained by PCs 1-3 (S3g Fig), PC 5 (0.9 % of variation) had the best correlation with LPS treatment alone (S3h Fig; Pearson r correlation coefficient=0.32). Principal component analysis (PCA) was performed using the PCA package in python from sklearn.decomposition. For protein PCA analysis, missing data (where no particles were detected) was substituted by the mean of that protein concentration for all other LPS treated or untreated samples. Pearson r was calculated using scipy.stats.pearsonr.

**ELISA analysis of protein concentrations**

Results of protein measurements were confirmed by enzyme-linked immunosorbant assay (ELISA). A549 cells from 4 biological replicates grown in separate flasks were seeded on a 24-well plate with each biological replicate receiving 6 wells. When cells reached ~80% confluency, cells were incubated for 24 hours with 10 µg/mL LPS following the protocol in the main paper. After 24 hours, supernatant was extracted and kept. Supernatant was kept for analysis at -80C. Cells were trypsinized for 10 minutes then centrifuged 5 minutes at 1500xg at room temperature, washed once with PBS, then centrifuged again for 5 minutes at 1500xg at room temperature. Supernatant was removed and cells were incubated in 1X cell lysis buffer diluted with water (ThermoFisher Scientific; IOCLB1). Lysed cell extract was stored at -80C until use. ELISA was performed using DuoSet ELISA kits from R&D Systems (biotechne), and was performed using kits for TNF$\alpha$ (DY210-05), IL1β (DY201-05), CCL22 (DY336), CXCL5 (DY254-05), CXCL10 (DY266-05) and CXCL8 (DY208-05). ELISA was performed following manufacturer protocol, using undiluted supernatant/cell lysis extract as sample input. CXCL5 supernatant was diluted 1:100 in reagent diluent (1% BSA in PBS) following DuoSet kit protocol. ELISA measurements were performed on BioTek Cytation 7 imaging system (Agilent) using absorbance measurements at 450 (signal) and 540 (background) nm. Absorbance measurements were processed by subtracting background at 540 from signal at 450, then subtracting the zero measurement (reagent diluent added in place of sample) from each sample. Sample concentrations were interpolated using standards provided in the DuoSet kits and Sigmoidal 4PL regression analysis in GraphPad Prism 10 following instructions in DuoSet kits. Values below the minium were set at the minimum interpolatable value. Bioreplicate values for LPS treated and untreated samples were the average of the three technical replicates performed for each. Values for bioreplicates were then compared using paired Student’s t-test.

**REFERENCES**

1. Davies T, Fearn T. Back to basics: the principles of principal component analysis. Spectroscopy Europe. 2004;16(6):20.
